# Synergistic Enhancement of Thermal Conductivity and Dielectric Properties in Al_2_O_3_/BaTiO_3_/PP Composites

**DOI:** 10.3390/ma11091536

**Published:** 2018-08-26

**Authors:** Junlong Yao, Li Hu, Min Zhou, Feng You, Xueliang Jiang, Lin Gao, Qing Wang, Zhengguang Sun, Jun Wang

**Affiliations:** 1 School of Materials Science and Engineering, Wuhan Institute of Technology, Wuhan 430205, China; junlongyao@yahoo.com (J.Y.); hulidrifter@outlook.com (L.H.); hspike@sina.com (M.Z.); youfeng.mse@wit.edu.cn (F.Y.); sjtujxl@163.com (X.J.); 2 Department of Materials Science and Engineering, The Pennsylvania State University, University Park, PA 16802, USA; 3 Ministry-of-Education Key Laboratory for the Green Preparation and Application of Functional Materials, Hubei University, Wuhan 430062, China; sunshine@hubu.edu.cn; 4 School of Chemistry and Environmental Engineering, Jianghan University, Wuhan 430056, China; 5 State Key Laboratory of Environmental Aquatic Chemistry, Research Center for Eco-Environmental Sciences, Chinese Academy of Sciences, Beijing 100085, China; junwang@rcees.ac.cn

**Keywords:** thermal conductivity, dielectric properties, synergistic effect, polymer composites, ceramic

## Abstract

Multifunctional polymer composites with both high dielectric constants and high thermal conductivity are urgently needed by high-temperature electronic devices and modern microelectromechanical systems. However, high heat-conduction capability or dielectric properties of polymer composites all depend on high-content loading of different functional thermal-conductive or high-dielectric ceramic fillers (every filler volume fraction ≥ 50%, i.e., *f*_filler_ ≥ 50%), and an overload of various fillers (*f*_thermal-conductive_ _filler_ + *f*_high-dielectric_ _filler_ > 50%) will decrease the processability and mechanical properties of the composite. Herein, series of alumina/barium titanate/polypropylene (Al_2_O_3_/BT/PP) composites with high dielectric- and high thermal-conductivity properties are prepared with no more than 50% volume fraction of total ceramic fillers loading, i.e., *f*_fillers_ ≤ 50%. Results showed the thermal conductivity of the Al_2_O_3_/BT/PP composite is up to 0.90 W/m·K with only 10% thermal-conductive Al_2_O_3_ filler, which is 4.5 times higher than the corresponding Al_2_O_3_/PP composites. Moreover, higher dielectric strength (*E*_b_) is also found at the same loading, which is 1.6 times higher than PP, and the Al_2_O_3_/BT/PP composite also exhibited high dielectric constant ($\varepsilon_{r}$ = 18 at 1000 Hz) and low dielectric loss (tan δ ≤ 0.030). These excellent performances originate from the synergistic mechanism between BaTiO_3_ macroparticles and Al_2_O_3_ nanoparticles.

## 1. Introduction

Ever-increasing demands of modern microelectromechanical systems and high-temperature electronic and energy storage devices have stimulated the research and design on multifunctional polymer composites [1,2,3,4,5,6,7]. Although polymers such as polypropylene (PP) exhibit excellent processability, insulativity, and are low-cost [8,9,10], the lack of dielectric properties restricts their application in dielectric materials and electronic component fields [3,4,5]. In the meantime, dielectric ceramic/polymer composites have received special attention due to their excellent combination of processability and the high dielectric performance of polymer and ceramic [11,12]. However, the miniaturization of electronic devices and the low heat resistance of the polymer matrix often causes difficulty in heat dissipation [1,3], and weaken the operation’s reliability of polymer composites at high temperature. As such, it is crucial to design and prepare a high thermal-conductive and high dielectric-polymer composite that can solve the heat problem [1,3,9], and retain the ideal electrical or dielectric performances at desired levels [1,2,13].

To improve the thermal properties, high thermal conductivity fillers, such as aluminum oxide (Al_2_O_3_) [14,15], boron nitride (BN) [16,17,18,19], silicon nitride (Si_3_N_4_) [20,21], and aluminum nitride (AlN) [22,23] etc. are added into the polymer matrices. However, a number of studies indicate that higher thermal conductivity or excellent dielectric property is always accompanied by high filler content [24,25] such as high thermal conductivity filler Al_2_O_3_ [14,15,26] (*f*_thermal-conductive filler_ > 50%) or high dielectric filler [4] such as BaTiO_3_ (BT) ceramic [24,25,27] (*f*_dielectric filler_ > 50%) to achieve the corresponding properties, and over load of various fillers (*f*_fillers_ > 50%) will greatly reduce processability and mechanical properties of the composite [21,28,29,30], and limited its applications scope. As a result, the overall thermal conductivity, dielectric properties, and mechanical properties etc. of the polymer composite are significantly affected within the polymer composite.

Recently, the use of thermal-conductive polymer composites filled with various inorganic fillers is growing up. For enhancement of the thermal conductivity, the composites are filled with different-scale particle fillers, which can form thermal-conductive connect chains or nets [18,31] among various size particles [32,33] with their compact packed contacts.

For those high thermal conductivity composites filled with different-sized particle fillers, it is possible to obtain high dielectric properties simultaneously if the larger particle size fillers are replaced by the same-sized high dielectric ceramic fillers. Meanwhile, the high thermal conductivity can be retained at a low filler content.

In addition, BaTiO_3_ and Al_2_O_3_ composites and devices have recently been reported as novel metamaterials [34,35,36,37,38], which can be applied in optical cloaking [39] and biochemical sensing [36,40,41,42,43,44] and detection [41,42], protein analysis [45], food quality analysis, and telecommunication [46], etc. They can also be used in tumor treatment, and radar cross-section reduction [46,47] as a therapeutic tool or a polarization conversion device due to their unique dielectric and electromagnetic properties [48]. This paper is focused on the enhancement of both the thermal conductivity and dielectric properties in series of alumina/barium titanate/polypropylene (Al_2_O_3_/BT/PP) composites with various volume ratios of Al_2_O_3_ and BT. It is found that the highest thermal conductivity of the composites could be observed when the volume fraction of high thermal conductive filler is at a low level ($f_{{\mathrm{Al}}_{2}O_{3}}$ = 10%). It is 4.5 times higher than that of the counterpart Al_2_O_3_/PP composites, and 5.3 times higher than that of pure PP. Improvement is achieved by the synergistic effect [33] in different-sized Al_2_O_3_ and BT particles in one polymer matrix. Meanwhile, higher dielectric properties are also obtained in the composite. The experimental results reveal a novel approach for the design and preparation of multifunctional polymer composites with different fillers, open many new research areas and lead to lots of novel applications in electromagnetic, optical cloaking [49] with metamaterials [50].

## 2. Materials and Methods

### 2.1. Materials

Polypropylene (PP) powder is prepared via the dissolution/precipitation method and is supplied by the Samsung Total Company HJ730, Seoul, Korea. Al_2_O_3_ is purchased from Aladdin Industrial Corporation, Shanghai, China and is used as soon as it is received. BaTiO_3_ (BT), which is usually used to improve the properties of dielectric, is supplied by Dianyang Company, Shanghai, China. The physical properties of Al_2_O_3_, BT and PP powders used in this study are given in Table 1.

### 2.2. Preparation of PP Powders

The PP granules are produced by dissolving in the dimethylbenzene solvent with a mass ratio of 1:40. The solvent is kept stirring for 3 h at 160 °C, and then cooled in the oven at 70 °C. Finally, the PP powders are achieved.

### 2.3. Preparation of Composite Sample

The Al_2_O_3_/BT/PP, Al_2_O_3_/PP, and BT/PP composites, 12 mm in diameter and 1.2–2 mm in thickness, are prepared from PP, Al_2_O_3,_ or BT powders by adopting a simple blending and hot-pressing procedure at about 170–190 °C and 15–20 MPa for 30 min in a die. Finally, the samples are coated with a silver paste.

### 2.4. Characterizations

A C-THERM Thermal Conductivity Analyzer is used to measure the out-of-plane thermal conductivity of cylindrical samples of Al_2_O_3_/BT/PP composites, which are 12 mm in diameter and 1.2–2 mm in thickness.

The values of dielectric properties and capacitance of the composites are obtained by using an Agilent-4980A impedance analyzer. Frequency range [51] is 100 Hz–2 MHz at an average voltage of 0.5 V. The relative dielectric constant ($\varepsilon_{r}$) is calculated according to Equation (1):

(1) $$\varepsilon_{r}=\frac{C\times d}{\varepsilon_{0}\times A}$$

*C* is the capacitance of the composite, *d* is the thickness of the discs (m), $\varepsilon_{0}$ is the vacuum dielectric constant (8.854 × 10^−12^ F·m^−1^), and A is the area of the major disc surface (m^2^).

The Maxwell Garnett Equation (2) [52] and logarithmic mixing Equation (3) [53] is used for calculating the theoretical dielectric constant in 0–3 type polymer composites

(2) $$\varepsilon_{\mathrm{eff}}=\varepsilon_{1}+3f\varepsilon_{1}\frac{\varepsilon_{2}-\varepsilon_{1}}{\varepsilon_{2}+2\varepsilon_{1}-f\left(\varepsilon_{2}-\varepsilon_{1}\right)}$$

where *f* represents volume fraction of the filler in the composite, while $\varepsilon_{2}$ and $\varepsilon_{1}$ are the dielectric constants of the filler and the polymer matrix, respectively,

log *ε* = log *ε*_polymer_ + *f*_filler_ log(*ε*_filler_/*ε*_polymer_) (3)

where *f*_filler_ represented volume fraction of the filler in the composite, *ε*_filler_ and *ε*_polymer_ are the dielectric constants of the filler and the polymer matrix, respectively.

The cross-sectional morphology pictures are made with a Japanese JEOL Hitachi S-530 scanning electron microscope (SEM, Japan Hitachi, Tokyo, Japan) at an acceleration voltage of 3 kV and a magnification of 5000 times and 10,000 times respectively. The images of cross-section of the composites are compared. The sample is coated with a conductive adhesive in the metal sample stage.

The breakdown strength measurement is conducted by using a dielectric strength tester (made by DH, Shanghai Lanpotronics Co., Shanghai, China). It is performed at room temperature using a DC voltage. Test voltage with ramp rate 2 V/s is applied.

## 3. Results and Discussion

### 3.1. Thermal Conductivity of the Composites

**Figure 1:**
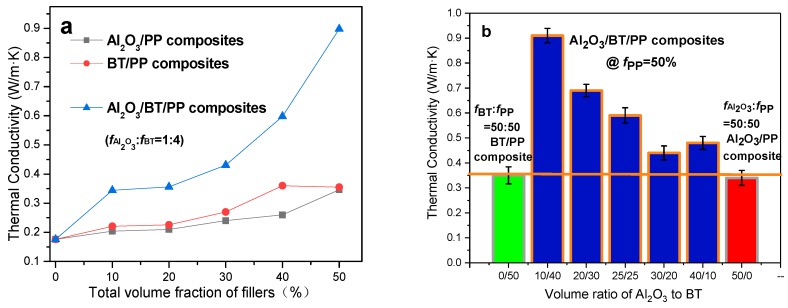
Thermal conductivity of the composites: (**a**) Al_2_O_3_/PP, BT/PP and Al_2_O_3_/BT/PP composites; (**b**) Al_2_O_3_/BT/PP composites.

Mostly PP composites or organic/inorganic blends are able to keep good mechanical performance with their total filler content *f*_fillers_ ≤ 50%, which has been reported in previous papers [28,29,30], therefore, the PP volume fraction is held as a constant at 50% (*f*_PP_ = 50%) in our experiments for maintaining a desirable processability and the mechanical properties, and a series of Al_2_O_3_/BT/PP, Al_2_O_3_/PP and BT/PP composites with various volume fractions of Al_2_O_3_ and BT particles are prepared. Thermal conductivities of these composites are shown in Figure 1a,b. As for Al_2_O_3_/PP or BT/PP binary composites, the thermal conductivities had been at a low level (≤0.35 W/m·K, $f_{{\mathrm{Al}}_{2}O_{3}}$ or *f*_BT_ ≤ 50%) before volume fraction of Al_2_O_3_ (or BT) particles increased from 10% to 50%. While comparing BT/PP and Al_2_O_3_/PP composites with same volume fraction, an abnormal thermal conductivity is found: the BT/PP composites actually present a relatively higher thermal conductivity (differ by no more than 0.10 W/m·K) than that of the Al_2_O_3_/PP composite, which is highly thermal-conductive.

In addition, the evaluation of the heat-conducting property of Al_2_O_3_/BT/PP composites yielded remarkable results: the thermal conductivity of Al_2_O_3_/BT/PP ternary composites is significantly higher (0.45–0.90 W/m·K, Figure 1b) compared with Al_2_O_3_/PP or BT/PP binary composites (0.17–0.35 W/m·K), and increased with the decrease of $f_{{\mathrm{Al}}_{2}O_{3}}$, i.e., the lower the $f_{{\mathrm{Al}}_{2}O_{3}}$ is, the higher the thermal conductivity is. And it is also found that the Al_2_O_3_/BT/PP composites reached its maximum thermal conductivity value (0.90 W/m·K) with lowest thermal conductive filler content ($f_{{\mathrm{Al}}_{2}O_{3}}$ = 10%). This maximum value was 5.3 times higher than that of pure PP (0.17 W/m·K), and 4.5 times higher than its counterpart Al_2_O_3_/PP composite (0.20 W/m·K, $f_{{\mathrm{Al}}_{2}O_{3}}$:*f*_PP_ = 10:90). The obviously synergistic effect on thermal conductivity is found in all the Al_2_O_3_/BT/PP composites (Figure 1b) with different ratios, and the lower the *f*_thermal-conductive filler_, the higher the thermal conductivity.

### 3.2. The Micrographs of Al_2_O_3_/BT/PP, Al_2_O_3_/PP and BT/PP Composites

**Figure 2:**
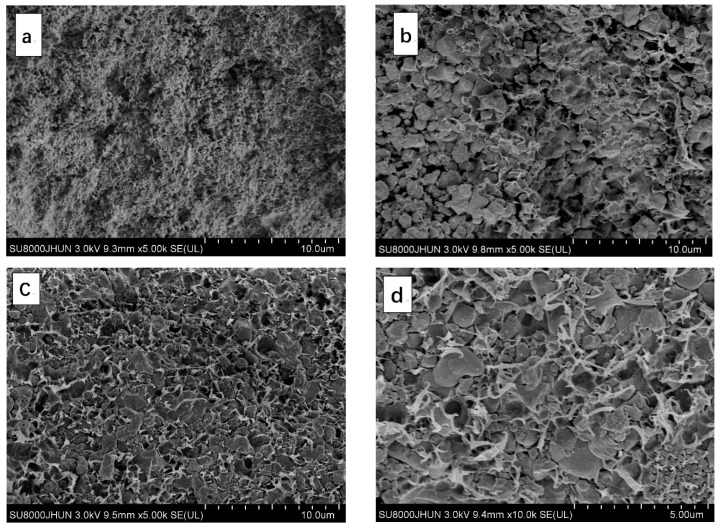
SEM micrographs of the fracture cross-section of the composites: (**a**) $f_{{\mathrm{Al}}_{2}O_{3}}$:*f*_PP_ = 50:50 Al_2_O_3_/PP composite; (**b**) *f*_BT_:*f*_PP_ = 50:50 BT/PP composite; (**c**,**d**) $f_{{\mathrm{Al}}_{2}O_{3}}$:*f*_BT_:*f*_PP_ = 10:40:50 Al_2_O_3_/BT/PP composite in different magnifications.

The thermal conductivity of polymer matrix composites are affected not only by the inherent thermal properties, but also by many other factors, such as shape, size, distribution, particle content and composite microstructure [33]. The mixed fillers with different volume ratios and particle sizes are used in this study, which may develop into unusual microstructures in Al_2_O_3_/BT/PP, Al_2_O_3_/PP and BT/PP composites [54]. Figure 2 shows the SEM images of the fracture cross-section of Al_2_O_3_/BT/PP ($f_{{\mathrm{Al}}_{2}O_{3}}$:*f*_BT_:*f*_PP_ = 10:40:50), Al_2_O_3_/PP ($f_{{\mathrm{Al}}_{2}O_{3}}$:*f*_PP_ = 50:50) and BT/PP composites (*f*_BT_:*f*_PP_ = 50:50).

In Figure 2a, nanoscale Al_2_O_3_ thermal-conductive particles are dispersed uniformly in the PP matrix. However, the thermal insulation polymer—PP—blocks the interconnection among Al_2_O_3_ particles and thus breaks up the extension of thermal conductive chains. On the contrary, from the micrograph of BT/PP composite in Figure 2b, it can be seen that although large number of voids and holes existed in low thermal-conductive BT/PP composites, the micrometer-scale BT particles, which are bigger than Al_2_O_3_ particles, make it easier for them to touch each other to form a heat conducting network [55], which leading to relatively higher thermal conductivity of BT/PP composites than that of Al_2_O_3_/PP composites. These results suggest the compact connection or arrangement of filler particles is more advantageous to improve thermal conductive property.

A stronger phenomena can be seen from Figure 2c, which shows the micromorphology of Al_2_O_3_/BT/PP composite ($f_{{\mathrm{Al}}_{2}O_{3}}$:*f*_BT_:*f*_PP_ = 10:40:50), where the voids and holes in the BT/PP composite disappeared. Instead, more homogeneous dispersion and more compact interface connection of BT and Al_2_O_3_ particles in PP matrix are observed.

**Figure 3:**
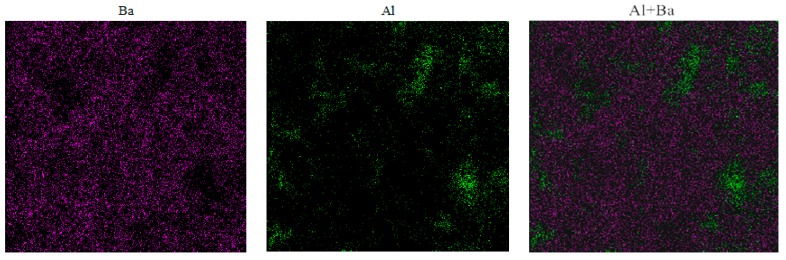
The EDS of Al, Ba and Al + Ba elemental in $f_{{\mathrm{Al}}_{2}O_{3}}$:*f*_BT_:*f*_PP_ = 10:40:50 Al_2_O_3_/BT/PP composite.

Further investigation of magnified Al_2_O_3_/BT/PP SEM micrograph (Figure 2d) indicate that the BT macroparticles are able to break down Al_2_O_3_ nanoparticle aggregations, thereby allowing Al_2_O_3_ nanoparticle to form the homogeneous dispersion with smaller size in a polymer matrix, and surround the big BT particles. The dispersion uniformity of Al_2_O_3_ and BT particles is verified again using energy dispersive spectrometer (EDS) of Al and Ba elementals. As shown in Figure 3, the Ba and Al element distribution diagrams demonstrated the uniform dispersion of plenty of BT particles in the polymer matrix, and the Al element formed many curves and nets at the same time; in the overlap of the two images, the compact structure between Al_2_O_3_ and BT particles are observed.

**Figure 4:**
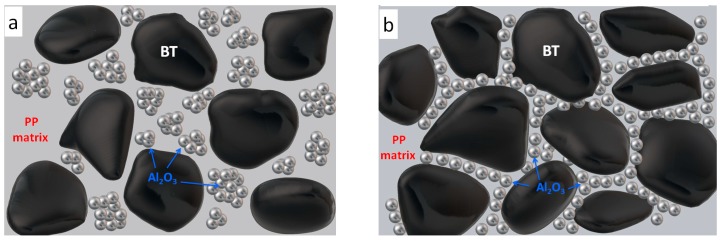
The schematic diagram of synergistic mechanism of thermal conductivity in *f*_PP_ = 50% Al_2_O_3_/BT/PP composites: (**a**) *f*_BT_:$f_{{\mathrm{Al}}_{2}O_{3}}$ = 40:10; (**b**) *f*_BT_:$f_{{\mathrm{Al}}_{2}O_{3}}$ < 40:10.

The synergistic mechanism of thermal conductivity in Al_2_O_3_/BT/PP composite is further illustrated in Figure 4. When *f*_BT_ is less than 40%, the holes and voids among BT particles are large enough. It leads to the loose distribution of Al_2_O_3_ nanoparticles and their agglomerations, which are easily wrapped up by heat-insulated PP matrix (Figure 4a), and cause lower thermal conductivity as shown in Figure 1b and Figure 4b. As the content of the BT particles increased, voids and holes between BT particles became narrower and smaller, and the smaller thermal-conductive nano-Al_2_O_3_ particles are forced to be directionally arranged and disperse along the fixed voids formed by BT macroparticles. As a result, many slim and compact Al_2_O_3_ thermal conductive chains and nets are formed.

When the ration of *f*_BT_:$f_{{\mathrm{Al}}_{2}O_{3}}$ increased to 40:10, the thermal conductivity further increase and reached the highest value due to more compact connection between thermal conductive Al_2_O_3_ nanoparticles with smaller size. Therefore, the thermal conductivity of Al_2_O_3_/BT/PP composites is greatly enhanced even at a lower thermal conductive filler content (Figure 4a), i.e., synergistic effect, and further demonstrated that the compact and directional connection of filler particles played a key role in improving the composite thermal conductivity.

### 3.3. Dielectric Properties of the Composites

**Figure 5:**
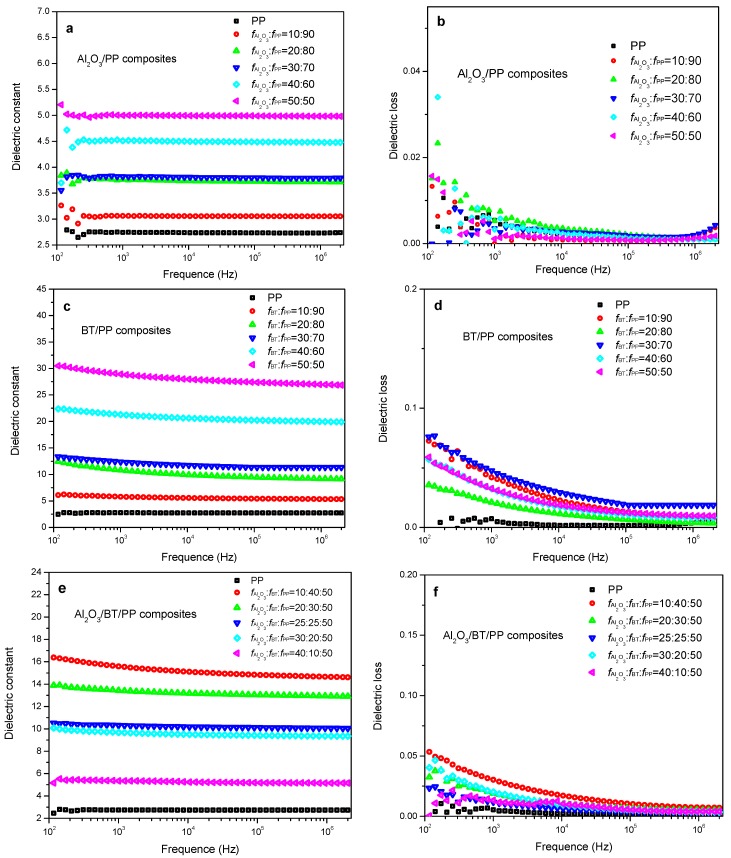
Dielectric constant/loss-frequency curves of Al_2_O_3_/PP, BT/PP and Al_2_O_3_/BT/PP composites from 100 Hz to 2 MHz: (**a**,**b**) Al_2_O_3_/PP composites; (**c**,**d**) BT/PP composites; and (**e**,**f**) Al_2_O_3_/BT/PP composites.

**Figure 6:**
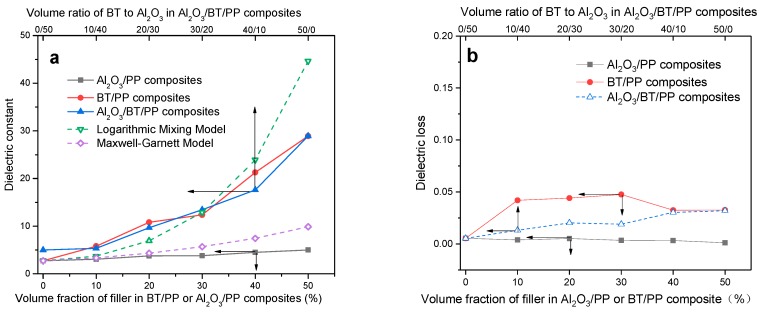
Dielectric properties of Al_2_O_3_/BT/PP, Al_2_O_3_/PP and BT/PP composites with different *f*_filler_ @ 1000 Hz: (**a**) dielectric constant of the composites; (**b**) dielectric loss of the composites.

Dielectric properties are closely related to the filling content and distribution [54] of dielectric particles in ceramic/polymer composites. As shown in Figure 5, the dielectric constant of Al_2_O_3_/PP, BT/PP and Al_2_O_3_/BT/PP composites increase with filler content; in Figure 6a,b, the dielectric constant ($\varepsilon_{r}$, at 1000 Hz) of Al_2_O_3_/BT/PP composites increased in accordance with BT volume, reaching the highest value of $\varepsilon_{r}$ = 18 at *f*_BT_ = 40%, which is higher than that of corresponding Al_2_O_3_/PP composites and pure PP ($\varepsilon_{r}\;$ < 5.0), and there is no obviously decrease in $\varepsilon_{r}$ compared with corresponding BT/PP composites ($\varepsilon_{r}\;$ = 20 at *f*_BT_:*f*_PP_ = 40:60). Meanwhile, the dielectric loss (tan, Figure 5f and Figure 6b) of Al_2_O_3_/BT/PP composites keep at a lower level (tan δ < 0.030 at 1000 Hz) when compared with corresponding BT/PP composites (tan δ < 0.050 at 1000 Hz).

The Maxwell Garnett Equation (2) is selected as the theoretical model due to its wide application in BT-loaded cloaking metamaterials and 0–3 type connectivity BT/PP, however, the calculated dielectric constant value is much lower than the measured value (one-third of measured value at *f*_BT_:*f*_PP_ = 50:50, Figure 6a). On the other hand, the logarithmic mixing Equation (3) applied in BT/cyanoethylated cellulose composites is more suitable for 0–3 type high-dielectric polymer composites (Figure 6a). Although the measured results of BT/PP and Al_2_O_3_/BT/PP composites are slightly lower than the theoretical values of BT/PP, the measured dielectric constant of BT/PP and Al_2_O_3_/BT/PP composites fit the logarithmic mixing model well. A more exact model for multiphase high-dielectric polymer composites will be discussed in later work.

High dielectric constant and low dielectric loss are simultaneously observed in Al_2_O_3_/BT/PP composites, and these optimal dielectric properties are attributed to high filling volume and uniform dispersion of BT particles, from the synergistic mechanism between BT macroparticles and Al_2_O_3_ nanoparticles. The presented material is facilely prepared using simply hot-express technology, and exhibit higher dielectric constant and higher thermal conductivity simultaneously, that urgently needed in modern energy storage and high temperature dielectric devices.

### 3.4. Breakdown Strength of the Composites

**Figure 7:**
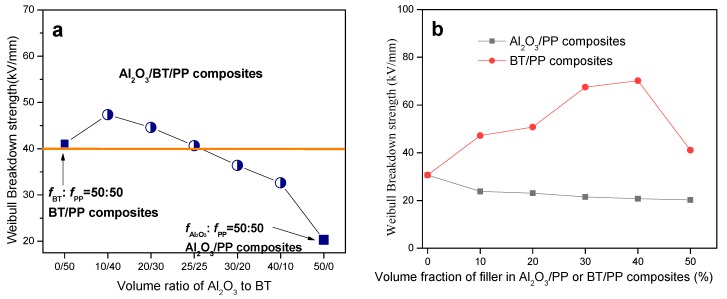
Weibull breakdown strength of the composites as a function of filler content: (**a**) Al_2_O_3_/BT/PP composites; (**b**) Al_2_O_3_/PP and BT/PP composites.

For dielectric and insulate polymer composites, breakdown strength (*E*_b_) could largely affect their operating electric field [56,57], which was influenced by many factors, including the *E*_b_ of filler and polymer matrix, and the distribution of filler particles and their interconnection in composites. Figure 7a,b shows the Weibull breakdown strength of the composites with different filler content. It can be seen that the addition of BT particles improved the breakdown strength of the composites, while the Al_2_O_3_ particles played an opposite role.

Although the *E*_b_ of BT is lower than that of Al_2_O_3_, the composites *E*_b_ mainly depend on the BT and Al_2_O_3_ particles distribution and interconnection, and this abnormal phenomenon is attributable to the more compact interconnection and homogeneous dispersion of Al_2_O_3_ and BT particles in polymer matrix.

For Al_2_O_3_/BT/PP composites, the value of Weibull *E*_b_ increase with the content of BT, and reached its peak value about 47.35 kV/mm when *f*_BT_:$f_{{\mathrm{Al}}_{2}O_{3}}$ = 40:10. It is higher than that of both Al_2_O_3_/PP composites (20.28 kV/mm, *f*_BT_:$f_{{\mathrm{Al}}_{2}O_{3}}$ = 50:50) and BT/PP composites (41.11 kV/mm, *f*_BT_:*f*_PP_ = 50:50), and even 1.6 times higher than pure PP (30.68 kV/m, Figure 7b). The voltage rise rate applied in this study is 2 V/s, the electric tree growing slowly inside the composites, and the compact microstructure and homogeneous dispersion of Al_2_O_3_ and BT particles (shown in Figure 2d) restrict the development of this process. So, a synergistic enhancement effect of the thermal conductivity, dielectric strength and dielectric constant is achieved at optimal $f_{{\mathrm{Al}}_{2}O_{3}}$:*f*_BT_:*f*_PP_ ratio of 10:40:50.

## 4. Conclusions

In this study, a series of fabricated Al_2_O_3_/BT/PP polymer composites emerged, which not only show significantly increased thermal conductivity but also higher dielectric properties with no more than 50% filling volume.

It was found that the thermal conductivity of the hybrid Al_2_O_3_/BT/PP composites could be enhanced significantly even when the thermal conductive fillers are kept at a lower volume fraction ($f_{{\mathrm{Al}}_{2}O_{3}}$ = 10%). The highest thermal conductivity reached 0.90 W/m·K, which is 4.5 times higher than that of Al_2_O_3_/PP composites and 5.3 times higher than that of pure PP. In addition, high dielectric strength (47.35 kV/mm), dielectric constant ($\varepsilon_{r}$ = 18 at 1000 Hz), and low dielectric loss (tan δ = 0.030) are also found in the Al_2_O_3_/BT/PP composite. This improvement came from the strong synergistic collaboration of particles with different sizes and their compact connection and directionally arrangement, making it possible to obtain both high thermal conductivity and high dielectric properties at low filler loading within one polymer matrix.

The experimental results could enhance the high temperature tolerance of PP dielectric materials in energy storage and in a supercapacitor, and revealed a novel approach for the preparation of organic/inorganic polymer composites with multi-function or -phase, which is crucial for the design of modern function composites, integrated electronic systems, and metamaterials [40,41,42,43,44,45,46,47]. The corresponding structure design and device will be further discussed and prepared in future.

## Figures and Tables

**Table 1:** The physical properties of Al_2_O_3_, BaTiO_3_ particles, and PP powder.

Materials	Average Particle Size (μm)	Density (g/cm^3^)	Dielectric Constant @ 1000 Hz	Breakdown Strength (kV/mm)	Thermal Conductivity (W/m·K)
Al_2_O_3_	0.1	4.00	10	15	42
BT	3	5.85	1000	3	6.2
PP	-	0.92	2.5	30	0.14
